# The GAUGAA Motif Is Responsible for the Binding between circSMARCA5 and SRSF1 and Related Downstream Effects on Glioblastoma Multiforme Cell Migration and Angiogenic Potential

**DOI:** 10.3390/ijms22041678

**Published:** 2021-02-07

**Authors:** Davide Barbagallo, Angela Caponnetto, Cristina Barbagallo, Rosalia Battaglia, Federica Mirabella, Duilia Brex, Michele Stella, Giuseppe Broggi, Roberto Altieri, Francesco Certo, Rosario Caltabiano, Giuseppe Maria Vincenzo Barbagallo, Carmelina Daniela Anfuso, Gabriella Lupo, Marco Ragusa, Cinzia Di Pietro, Thomas Birkballe Hansen, Michele Purrello

**Affiliations:** 1 Department of Biomedical and Biotechnological Sciences, Section of Biology and Genetics “Giovanni Sichel”, University of Catania, 95123 Catania, Italy; caponnettoangela@gmail.com (A.C.); barbagallocristina@gmail.com (C.B.); rosaliabattaglia04@gmail.com (R.B.); mirabella.federica.91@gmail.com (F.M.); duiliabrex@gmail.com (D.B.); michelestella7@gmail.com (M.S.); mragusa@unict.it (M.R.); dipietro@unict.it (C.D.P.); purrello@unict.it (M.P.); 2 Interdisciplinary Research Centre on the Diagnosis and Therapy of Brain Tumors, University of Catania, 95123 Catania, Italy; roberto.altieri.87@gmail.com (R.A.); cicciocerto@yahoo.it (F.C.); gbarbagallo@unict.it (G.M.V.B.); 3 Department of Medical, Surgical Sciences and Advanced Technologies “G.F. Ingrassia”, Section of Anatomic Pathology, University of Catania, 95123 Catania, Italy; giuseppe.broggi@gmail.com (G.B.); rosario.caltabiano@unict.it (R.C.); 4 Department of Medical, Surgical Sciences and Advanced Technologies “G.F. Ingrassia”, Neurological Surgery, Policlinico “Rodolico-San Marco” University Hospital, University of Catania, 95123 Catania, Italy; 5 Department of Biomedical and Biotechnological Sciences, Section of Medical Biochemistry, University of Catania, 95123 Catania, Italy; anfudan@unict.it (C.D.A.); lupogab@unict.it (G.L.); 6 Research Center for Prevention, Diagnosis and Treatment of Cancer, University of Catania, 95123 Catania, Italy; 7 Oasi Research Institute—IRCCS, 94018 Troina, Italy; 8 Department of Molecular Biology and Genetics (MBG), Aarhus University, 8000 Aarhus C, Denmark; tbh@mbg.au.dk; 9 Interdisciplinary Nanoscience Center (iNANO), Aarhus University, 8000 Aarhus C, Denmark

**Keywords:** circSMARCA5, RNA-binding proteins, SRSF1, VEGFA, SRSF3, binding site mutation, angiogenesis

## Abstract

Circular RNAs (circRNAs) are a large class of RNAs with regulatory functions within cells. We recently showed that circSMARCA5 is a tumor suppressor in glioblastoma multiforme (GBM) and acts as a decoy for Serine and Arginine Rich Splicing Factor 1 (SRSF1) through six predicted binding sites (BSs). Here we characterized RNA motifs functionally involved in the interaction between circSMARCA5 and SRSF1. Three different circSMARCA5 molecules (Mut1, Mut2, Mut3), each mutated in two predicted SRSF1 BSs at once, were obtained through PCR-based replacement of wild-type (WT) BS sequences and cloned in three independent pcDNA3 vectors. Mut1 significantly decreased its capability to interact with SRSF1 as compared to WT, based on the RNA immunoprecipitation assay. In silico analysis through the “Find Individual Motif Occurrences” (FIMO) algorithm showed GAUGAA as an experimentally validated SRSF1 binding motif significantly overrepresented within both predicted SRSF1 BSs mutated in Mut1 (*q*-value = 0.0011). U87MG and CAS-1, transfected with Mut1, significantly increased their migration with respect to controls transfected with WT, as revealed by the cell exclusion zone assay. Immortalized human brain microvascular endothelial cells (IM-HBMEC) exposed to conditioned medium (CM) harvested from U87MG and CAS-1 transfected with Mut1 significantly sprouted more than those treated with CM harvested from U87MG and CAS-1 transfected with WT, as shown by the tube formation assay. qRT-PCR showed that the intracellular pro- to anti-angiogenic Vascular Endothelial Growth Factor A (VEGFA) mRNA isoform ratio and the amount of total VEGFA mRNA secreted in CM significantly increased in Mut1-transfected CAS-1 as compared to controls transfected with WT. Our data suggest that GAUGAA is the RNA motif responsible for the interaction between circSMARCA5 and SRSF1 as well as for the circSMARCA5-mediated control of GBM cell migration and angiogenic potential.

## 1. Introduction

Circular RNAs (circRNAs) are a recently discovered class of RNAs (mostly non-coding), characterized by the covalent bond between their 3′ and 5′ ends and detected from archea to humans [1,2,3]. Notwithstanding the data on their expression profile, both in physiological and pathological conditions, and evidence on their involvement in several biological processes (from cell cycle control to epithelial-mesenchymal transition (EMT), etc.) are increasing day by day, biogenesis and function of circRNAs are still not fully characterized [4]. The expression of several circRNAs has been found to be altered in different cancers, where they may exert either an oncogenic or a tumor suppressive function, independent from their linear isoform counterparts [5,6,7,8]. CircRNAs have been mainly characterized as microRNA and RNA binding protein (RBP) sponges or decoys, involved in complex epigenetic competitive endogenous RNA (ceRNA) networks within cells [2,9,10,11,12,13].

Glioblastoma multiforme (GBM) is the most aggressive primary tumor of the central nervous system (CNS) [14]. The World Health Organization (WHO) classification of CNS tumors was revised in 2016 to incorporate molecular biomarkers, in addition to classic histological features [15]. Since then, a growing number of new genomic, epigenomic and radiogenomic data of GBM cells have been collected, creating the background for an updated classification of these tumors in the near future [16,17]. Dysregulation of circRNAs in GBM cells has been recently ascertained by our group as well as by other researchers [8,18,19,20,21,22].

Among circRNAs, we recently focused on circSMARCA5, a 269 nucleotide-long circRNA, abundantly expressed in the human brain in physiological conditions. CircSMARCA5 has been described as a tumor suppressor in several cancers [23,24,25,26,27] and we recently proposed that it acts as a tumor suppressor in GBM [8,18]. Our data obtained in GBM biopsies and U87MG GBM cell line showed that circSMARCA5: (i) is significantly downregulated in GBM biopsies, as compared to normal brain parenchyma; (ii) acts as a decoy for the oncogenic Serine and Arginine Rich Splicing Factor 1 (SRSF1); (iii) shows a negative correlation between its expression and the number of blood vascular microvessel density; (iv) acts as a regulator of the SRSF1-mediated splicing activity; and (v) shows a positive correlation between its expression and both overall patient survival and progression free survival [8,18].

SRSF1 is a known pleiotropic nucleus-to-cytoplasm shuttling protein, involved in the development of several human cancers [28,29]. More specifically, SRSF1 is known to contribute to GBM pathogenesis by either splicing-dependent mechanisms (e.g., by increasing the ratio of pro- to anti-oncogenic splicing isoforms of myosin IB (MYO1B) [30,31] and MAPK Signal-Integrating Kinase 2 (MKNK2) [32]) or splicing-independent mechanisms (e.g., by stabilizing the oncogenic nuclear paraspeckle assembly transcript 1 (NEAT1) [33] or by a competitive self-binding interaction that inhibits the targeting of its mRNA transcript from mir-505-3p [34]). Moreover, the involvement of SRSF1 post-translational regulator SRSF protein kinase 1 (SRPK1) in gliomagenesis has also been ascertained [35,36,37,38]. Accordingly, the interest in studying further the epigenetic regulation of SRSF1, through endogenous or exogenous RNA molecules acting as sponges, has been recently increasing [13,39]. In this scenario, we aimed to deepen the knowledge on circSMARCA5 by identifying its sequences functionally involved in the crosstalk with SRSF1.

## 2. Results

### 2.1. Generation of Wild Type (WT) and Mutated circSMARCA5 Constructs

**Figure 1:**
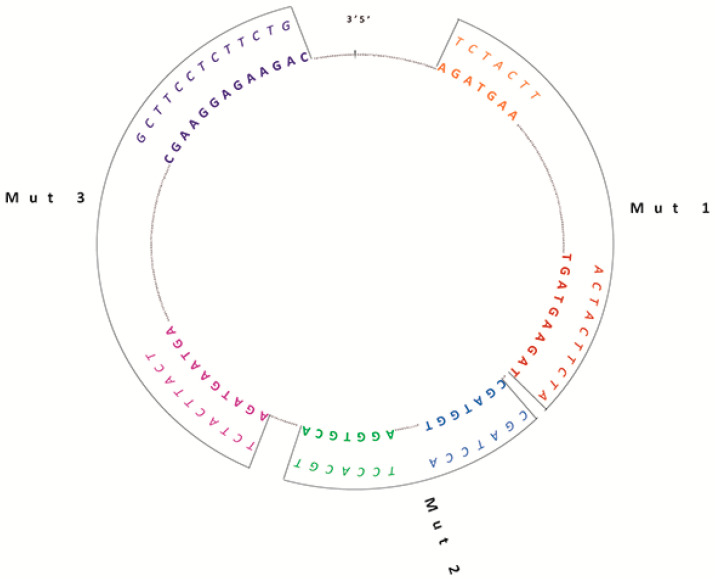
Schematic representation of wild-type (WT) and mutated circSMARCA5 sequences. The inner circle represents the whole WT circSMARCA5 sequence: predicted SRSF1 BSs are highlighted in bold, using different colors. In the outer arches mutated SRSF1 BSs are indicated in italics.

As previously reported by our group, we predicted six binding sites (BSs) for the splicing factor SRSF1 within the sequence of circSMARCA5 by querying the RBPMap database (http://rbpmap.technion.ac.il/) [18] (Figure 1). Based on these data, we cloned three independent mutated circSMARCA5 (Mut 1, Mut2, Mut 3) in three distinct pcDNA3 vectors, starting from the WT circSMARCA5 sequence. Each mutated circSMARCA5 harbored mutations in two out of the six predicted SRSF1 BSs at once (Table 1, Figure 1, Materials and Methods). Sequences of mutated circSMARCA5 were confirmed by Sanger sequencing. We also verified that each construct (harboring either WT or mutated circSMARCA5) synthesized the circular isoform of SMARCA5, as confirmed by northern analysis and qRT-PCR data (Figure S1).

### 2.2. RNA Immunoprecipitation (RIP) Assay Demonstrates That Mut1 circSMARCA5 Is Significantly Less Able to Interact with SRSF1 than WT, Mut2 and Mut3 circSMARCA5

**Figure 2:**
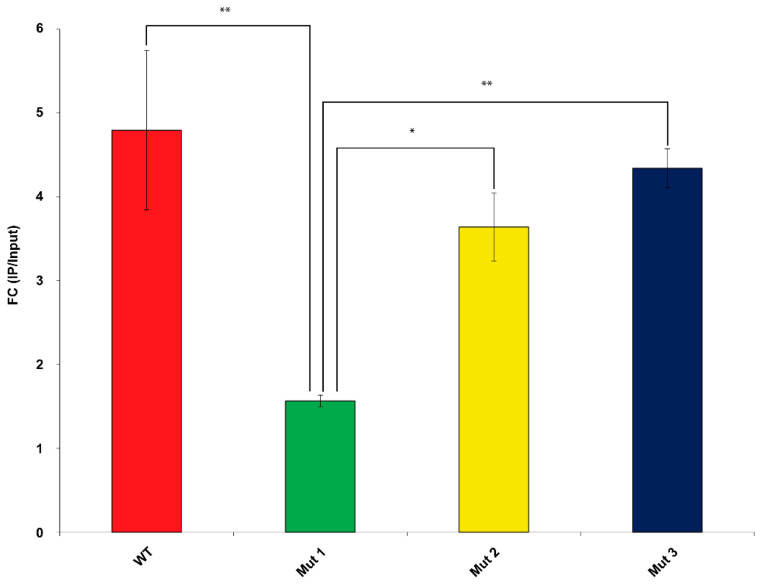
Mut1 circSMARCA5 shows a significantly reduced ability to interact with SRSF1 with respect to WT, Mut2 and Mut3 circSMARCA5. The bar graph represents the enrichment of WT, Mut1, Mut2 and Mut3 circSMARCA5 within U87MG cell lysates immunoprecipitated (IPed) with anti-FLAG antibody. Data are reported as mean ± standard deviation of fold-change (FC) of circSMARCA5 expression in FLAG-tagged IPed vs. mouse isotype control IgG IPed cell lysates, obtained from three independent experiments (see Supplementary Materials and Methods section). * *p*-value < 0.05, ** *p*-value < 0.01 (*n* = 3, One-way Analysis of Variance (ANOVA) with post-hoc Tukey Honestly Significant Difference (HSD) Test).

RIP performed on cell lysates obtained from U87MG co-transfected with FLAG-tagged SRSF1 and WT or mutated circSMARCA5 showed that Mut 1 circSMARCA5 significantly reduced its ability to interact with SRSF1 with respect to WT (more than three-fold), Mut2 and Mut3 (more than two-fold) circSMARCA5. Conversely, Mut2 and Mut3 circSMARCA5 did not show significant differences in their ability to interact with SRSF1, as compared to WT circSMARCA5 (Figure 2).

### 2.3. In Silico Analysis Predicts the GAUGAA Motif as Critically Involved in the Interaction between circSMARCA5 and SRSF1

The ATtRACT database revealed 445 RNA motifs, experimentally validated to interact with SRSF1: these motifs were used to train the “Find Individual Motif Occurrences” (FIMO) tool to find significant occurrences among the six predicted SRSF1 BSs. Two (UGAAGAU and UGAUGAA) motifs and one (GAAGGAG) motif significantly occurred (*q*-value = 0.0011) within predicted SRSF1 BSs mutated in Mut1 and Mut3 circSMARCA5, respectively (Table 2). The sequence GAUGAA, embedded in one of the three significantly occurring motifs, was enclosed within both predicted SRSF1 BSs mutated in Mut1 circSMARCA5.

### 2.4. U87MG and CAS-1 Transfected with Mut1 circSMARCA5 Migrate Significantly More than Controls Transfected with WT circSMARCA5

**Figure 3:**
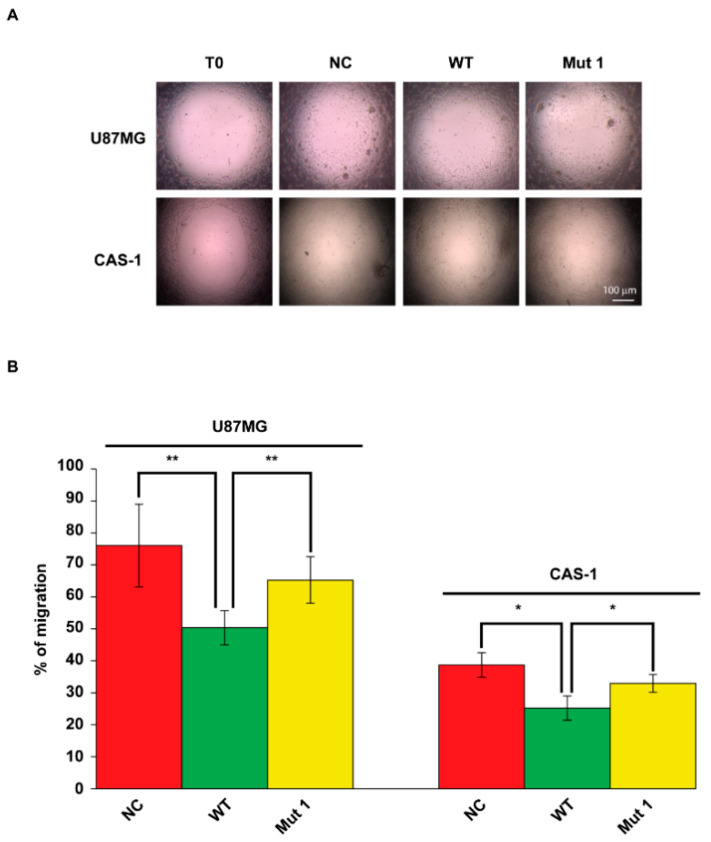
U87MG and CAS-1 transfected with Mut1 circSMARCA5 migrate significantly more than those transfected with WT circSMARCA5. (**A**) Representative micrographs of U87MG and CAS-1 migration at the starting point (T0) and at the endpoint (24 h and 32 h after stopper removal, respectively) in different experimental conditions: cells transfected with the empty pcDNA3 vector (NC), WT circSMARCA5 (WT), Mut1 circSMARCA5 (Mut1). (**B**) Bar graph showing the percentage (%) of U87MG and CAS-1 cell migration in the experimental conditions reported in (**A**). % of cell migration was calculated as follows, for each experimental condition: [(T0 Area—endpoint Area)/T0 Area] × 100%. * *p*-value < 0.05, ** *p*-value < 0.01 (*n* = 6, Student’s *t*-test). Data are reported as mean ± standard deviation of the % of cell migration, obtained from six independent experiments.

U87MG and CAS-1 transfected with Mut1 circSMARCA5 migrated significantly more (+15% and +8%, respectively) than those transfected with an equivalent amount of WT circSMARCA5 and did not show significant differences when compared with NC (Figure 3A,B). In the same experiment, U87MG and CAS-1 transfected with WT circSMARCA5 migrated significantly less (−26% and −13%, respectively) than cells transfected with the empty pcDNA3 vector (NC), confirming the data reported in our previous paper [8] (Figure 3A,B).

### 2.5. Conditioned Medium (CM) from U87MG or CAS-1 Transfected with Mut1 circSMARCA5 Increases Immortalized Human Brain Microvascular Endothelial Cell (IM-HBMEC) Sprouting as Compared to CM from U87MG or CAS-1 Transfected with WT circSMARCA5

**Figure 4:**
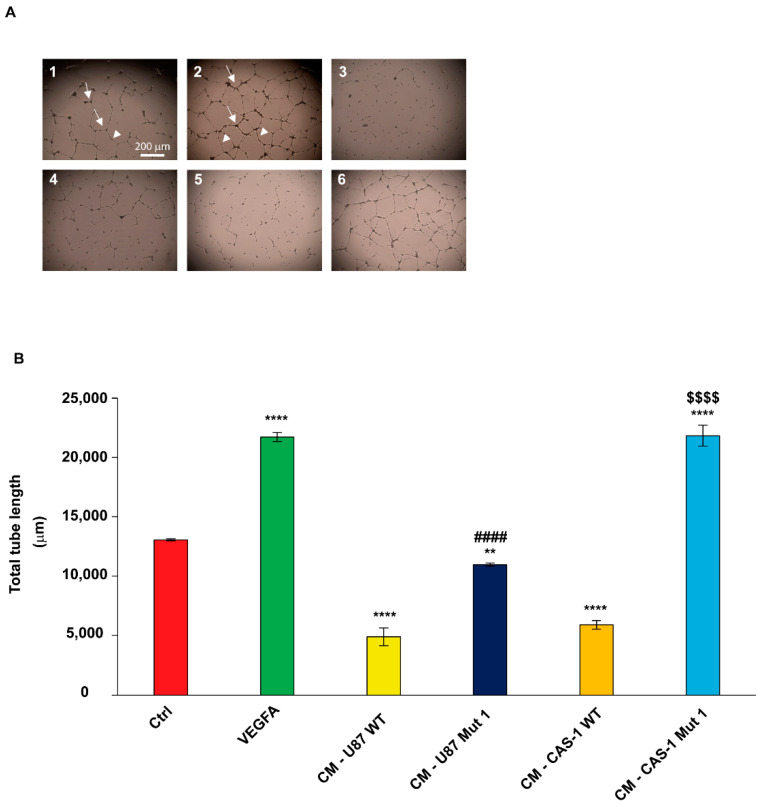

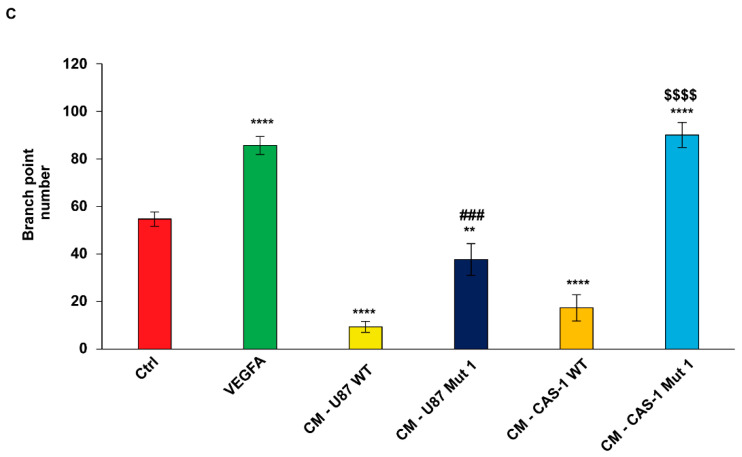
Conditioned medium (CM) from U87MG or CAS-1 transfected with Mut1 circSMARCA5 significantly increases IM-HBMEC cell sprouting as compared to CM from U87MG and CAS-1 transfected with WT circSMARCA5. (**A**) Representative optical phase-contrast micrographs (inverted light microscope, 40× magnification) showing IM-HBMEC cell-to-cell organization under different experimental conditions: (1) untreated IM-HBMEC (Ctrl); (2) IM-HBMEC grown in medium with the addition of 25 ng/mL recombinant human VEGF-A (VEGF-A 165 isoform) (VEGFA); (3) IM-HBMEC treated with CM harvested from U87MG transfected with WT circSMARCA5 (CM—U87 WT); (4) IM-HBMEC treated with CM harvested from U87MG transfected with Mut1 circSMARCA5 (CM—U87 Mut1); (5) IM-HBMEC treated with CM harvested from CAS-1 transfected with WT circSMARCA5 (CM—CAS-1 WT); (6) IM-HBMEC treated with CM harvested from CAS-1 transfected with Mut1 circSMARCA5 (CM—CAS-1 Mut1). Cell clusters emitted long offshoots (branch points, arrows; tube elongation, arrowheads) from cell bodies, essential for cells to mutually come into physical contact and to allow for spatial organization. (**B**) Bar graph representing the total tube length (µm) and (**C**) the branch point number of IM-HBMEC, under the same experimental conditions reported in (**A**). Data are represented as mean ± standard deviation of three independent experiments (n = 3). ** *p*-value < 0.01, **** *p*-value < 0.0001 vs. Ctrl; ### *p*-value < 0.001, #### *p*-value < 0.0001 vs. CM—U87 WT; $$$$ *p*-value < 0.0001 vs. CM—CAS-1 WT, One-way ANOVA with post-hoc Tukey HSD Test.

IM-HBMEC treated with CM harvested from U87MG (Figure 4A,B) or CAS-1 (Figure 4A,C) transfected with Mut1 circSMARCA5 showed a significant cell sprouting increase (both in terms of total tube length and number of branch points) with respect to controls (IM-HBMEC exposed to CM harvested from U87MG or CAS-1 transfected with WT circSMARCA5). The biological effect of CM from CAS-1 appeared to be more evident as compared to CM from U87MG: IM-HBMEC exposed to CM harvested from CAS-1 transfected with Mut1 circSMARCA5 increased their total tube length of 3.7-fold compared with controls (IM-HBMEC exposed to CM harvested from CAS-1 transfected with WT circSMARCA5); the observed increase of IM-HBMEC total tube length was of 2.2-fold in the case of CM from U87MG. The difference between the two fold-changes (FC) was statistically significant (*p*-value = 7.44778 × 10^−5^, Student’s *t*-test). In the same experiment, a significant increase (more than 1.5-fold, on average) of IM-HBMEC cell sprouting was observed after treatment with Vascular Endothelial Growth Factor A (VEGFA) (used as positive control of endothelial cell sprouting) as compared to untreated IM-HBMEC (Figure 4A–C); conversely, IM-HBMEC cell sprouting significantly decreased after treatment with CM from U87MG (Figure 4A,B) or CAS-1 (Figure 4A,C) cells transfected with WT circSMARCA5, as compared to untreated IM-HBMEC.

### 2.6. Pro- to Anti-Angiogenic VEGFA mRNA Isoform Ratio and the Amount of Extracellular Total VEGFA mRNA Increase in CAS-1 Transfected with Mut1 circSMARCA5 as Compared to CAS-1 Transfected with WT circSMARCA5

**Figure 5:**
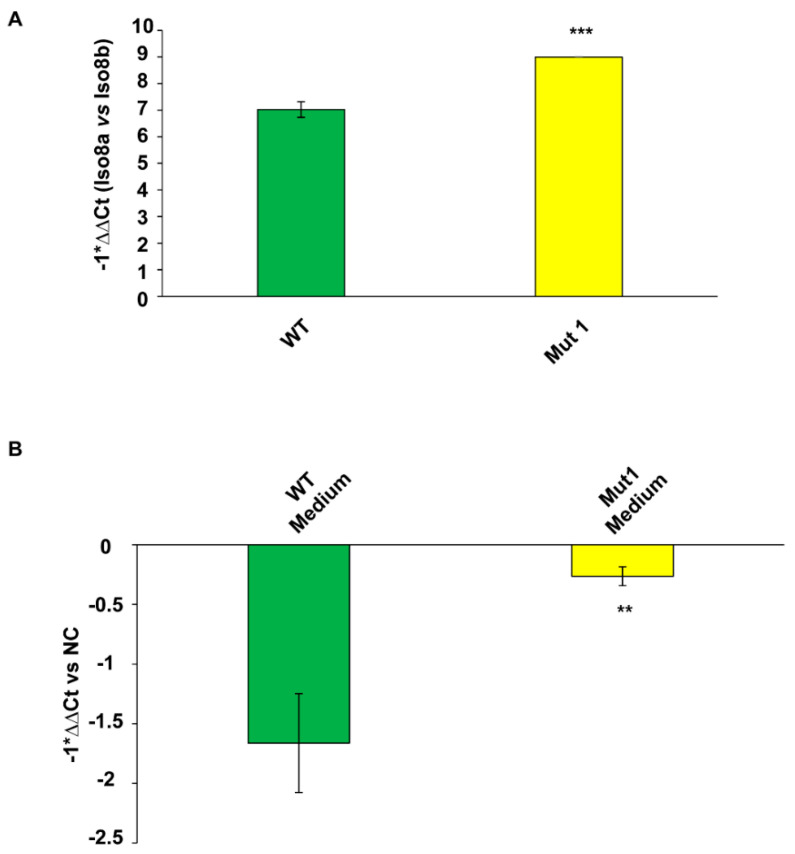
CAS-1 transfected with Mut1 circSMARCA5 show an increased (**A**) intracellular Iso8a to Iso8b VEGFA mRNA isoform ratio and (**B**) amount of secreted total VEGFA mRNA in cell culture medium as compared to CAS-1 transfected with WT circSMARCA5. (**A**) Data are represented as mean ± standard deviation of −1*ΔΔCt (the lower the value, the lower the relative expression of intracellular Iso8a vs. Iso8b VEGFA mRNA isoform; vice versa, the higher the value, the higher the intracellular Iso8a to Iso8b mRNA isoform ratio). ∆Ct values of each mRNA isoform were calculated from qRT-PCR experiments by using TATA-box binding protein (TBP) mRNA as endogenous control. ∆∆Ct was obtained subtracting ∆Ct of Iso8b to ∆Ct of Iso8a. (**B**) Data are represented as mean ± standard deviation of −1*ΔΔCt (the lower the value, the lower the expression of total VEGFA mRNA in cell culture medium; vice versa, the higher the value, the higher, the expression of total VEGFA mRNA in cell culture medium). Actin beta (ACTB) mRNA was used as endogenous control to calculate ∆Ct. Cell culture medium from CAS-1 transfected with an empty pcDNA3 vector (negative control, NC) was used as a calibrator to calculate ∆∆Ct. *** *p*-value < 0.001, ** *p*-value < 0.01 (*n* = 3, Student’s *t*-test vs. WT).

Based on tube formation assay data, we focused on CAS-1 to see how the lack of binding between SRSF1 and Mut1 circSMARCA5 impacts on VEGFA mRNA expression. CAS-1 transfected with Mut1 circSMARCA5 increased both the intracellular pro (Iso8a)- to anti (Iso8b)-angiogenic VEGFA mRNA isoform ratio and the amount of total VEGFA mRNA released in cell culture medium with respect to the controls (CAS-1 transfected with WT circSMARCA5) (Figure 5A,B, Figure S2). In silico analysis performed by Basic Local Alignment Search Tool (BLAST) allowed us to exclude amplification of possible traces of fetal bovine VEGFA mRNA contained in cell culture medium, by using our primer pairs.

### 2.7. Interplay between circSMARCA5, Its Negative Regulator DExH-Box Helicase 9 (DHX9), and VEGFA

**Figure 6:**
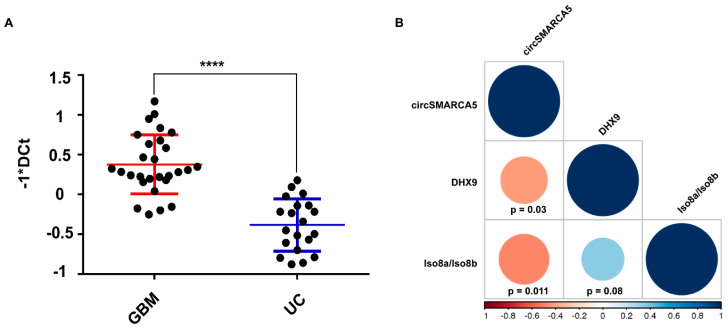
DHX9 mRNA is upregulated in GBM tumor biopsies and its expression negatively correlates with that of circSMARCA5. (**A**) Column scatter dot plot graph representing the expression of DHX9 mRNA in a cohort of GBM tumor biopsies (*n* = 28) and unaffected brain parenchyma (UC) (*n* = 20) (**** *p*-value < 0.0001, Student’s *t*-test). Expression data are represented as −1*DCt (the higher the value, the higher the expression of DHX9 mRNA; vice versa, the lower the value, the lower the expression of DHX9 mRNA). TBP mRNA was used as endogenous control to calculate ∆Ct. (**B**) Matrix of correlations among the expression of DHX9 mRNA, circSMARCA5, and Iso8a to Iso8b VEGFA mRNA isoform ratio (Iso8a/Iso8b). Blue and red color indicate positive and negative correlations, respectively. Correlation coefficients (r-values) are shown in the color scale bar. Size of the circle and their color intensity are proportional to the r-values in the correlation matrix.

In order to extend the pathway regulated by circSMARCA5 to upstream signals, we investigated the expression of: (i) circSMARCA5, (ii) mRNA of its negative regulator DHX9 (see Discussion), and (iii) Iso8a and Iso8b VEGFA mRNA isoforms in a cohort of 28 GBM tumor biopsies. DHX9 mRNA was significantly upregulated both in GBM biopsies (Figure 6A) and GBM cell lines (CAS-1 and U87MG) (Figure S3) with respect to controls. DHX9 mRNA expression negatively correlated with that of circSMARCA5 and positively correlated with the Iso8a to Iso8b VEGFA mRNA isoform ratio (Figure 6B). These data were confirmed in a larger cohort from TCGA (Figure S4).

## 3. Discussion

**Figure 7:**
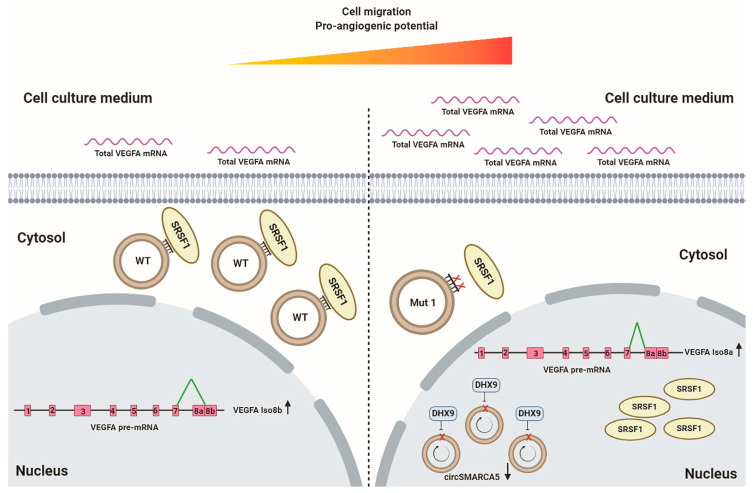
Schematic representation of the proposed mechanism of action of WT and Mut1 circSMARCA5 within GBM cells. Left panel: SRSF1 pro-oncogenic activity is negatively affected in GBM cells overexpressing WT circSMARCA5. The latter acts as a decoy for SRSF1, leading to (i) an increased relative expression of Iso8b vs. Iso8a VEGFA mRNA isoform; (ii) a decreased GBM cell migration and pro-angiogenic potential; (iii) a decreased secretion of total VEGFA mRNA in GBM cell culture medium. Right panel: Mut1 circSMARCA5 is less able to bind SRSF1 than WT circSMARCA5. The decreased decoy activity of Mut1 circSMARCA5 against SRSF1 causes the increase of (i) relative expression of Iso8a vs. Iso8b VEGFA mRNA isoform; (ii) GBM cell migration and pro-angiogenic potential; (iii) the secretion of total VEGFA mRNA in GBM cell culture medium. The biological effects shown in GBM cells overexpressing Mut1 circSMARCA5 resemble the dysregulation of the molecular axis involving circSMARCA5 (downregulated) and its negative regulator DHX9 (upregulated), observed within GBM biopsies. Red boxes indicate exons, black lines indicate introns, and green lines indicate possible splicing patterns leading to the recognition of a canonical (Iso8a) or an alternative (Iso8b) 3′ splice site within VEGFA pre-mRNA.

CircRNA functioning within cells is a relatively new, exciting and expanding research field [40,41]. Much more knowledge on the mechanics of the interaction between circRNAs and their molecular partners is still to be discovered. Here we deepen the knowledge on circSMARCA5 through in vitro and in silico characterization of its sequences regulating the interaction with SRSF1 and some related biological functions in GBM cells. Our data allowed to identify GAUGAA as the RNA motif critical for the interaction between circSMARCA5 and SRSF1: its mutation, occurring twice within Mut1 circSMARCA5, may indeed explain the decreased ability of this mutated circRNA to bind SRSF1 with respect to WT, Mut2 and Mut3 circSMARCA5. Our results are in agreement with other literature data showing that SRSF1 preferentially binds GA-rich RNA sequences [42,43]. Furthermore, our data are in line with the finding that the effect of a decoy RNA oligonucleotide positively correlates with the number of protein binding motif repeats embedded within its sequence [39,44]. The specific structural constraints of circRNAs and the critical role played by secondary and tertiary RNA structures in the binding with RBPs are known [45,46,47]: in this context, the identified interaction between circSMARCA5 and SRSF1 may be considered specific, strong and of interest for the study of the molecular bases of GBM. The decreased ability of Mut1 circSMARCA5 to bind SRSF1 entailed an increased GBM cell migration rate and pro-angiogenic potential, coupled to a higher pro- to anti-angiogenic VEGFA mRNA isoform ratio and increased amount of secreted extracellular VEGFA mRNA. These data are in line with our previous findings on GBM, where we showed that circSMARCA5 is a downregulated tumor suppressor: its downregulation contributed to its decreased ability to act as a decoy for SRSF1, a known oncoprotein, aberrantly expressed in several cancers [8,18,48,49,50]. Upregulation of DHX9 (a helicase known to generally suppress circRNA biogenesis [51]) mRNA and negative correlation between its expression and that of circSMARCA5 in GBM biopsies led us to hypothesize its activity as negative regulator of circSMARCA5 expression (Figure 7). Our hypothesis is in agreement with the data reported by Yu and colleagues that demonstrated how circSMARCA5 biogenesis is controlled by DHX9 in hepatocellular carcinoma [52]. The increase of IM-HBMEC sprouting after treatment with CM from CAS-1 transfected with Mut1 circSMARCA5 is in line with the increase of total VEGFA mRNA secreted by CAS-1 in the same CM. Secretion of mRNAs in tumor cell CM has been described by O’Driscoll and colleagues [53]. Contribution of secreted full-length mRNA in GBM tumor progression [54] and the release of extracellular vesicles containing pro-angiogenic functional cargo, comprising VEGFA mRNA, by GBM cells have also been ascertained [55,56,57,58]. In the near future, the use of circular RNA aptamers, appropriately designed to restore the circSMARCA5 pathway, may be assayed as therapeutic agents against GBM [39,44,59,60].

## 4. Materials and Methods

### 4.1. Cell Culture

U87MG were purchased from American Type Culture Collection (ATCC) and cultured in Dulbecco’s Modified Eagle’s Medium (DMEM), 1 g/L glucose (DMEM, Sigma-Aldrich, Milan, Italy), supplemented with 10% fetal bovine serum (FBS, ThermoFisher Scientific, Gibco™, Waltham, MA, USA), 1% penicillin and streptomycin (PAN-Biotech, Aidenbach, Bavaria, Germany), 1% non-essential amino acids (Dominique Dutscher SAS, Alsace, France), 1% sodium pyruvate (ThermoFisher Scientific), at 37 °C in a humidified 95% air and 5% CO_2_ atmosphere. The human GBM cell line CAS-1 (ICLC, Genova, Italy) was cultured in DMEM, 1 g/L glucose (Sigma-Aldrich, Milan, Italy) supplemented with 10% FBS (ThermoFisher Scientific, Gibco™), 1% penicillin and streptomycin (PAN-Biotech,), 2 mM l-Glutamine (ThermoFisher Scientific, Gibco™), at 37 °C in a humidified 95% air and 5% CO_2_ atmosphere. IM-HBMEC (Innoprot, Elexalde Derio, Spain) were grown as a monolayer in endothelial cell medium (ECM) (ScienCell Research Laboratories, Carlsbad, CA, USA. Cat. No. 1001), supplemented with 5% (*v*/*v*) FBS (Sigma-Aldrich Merck, Cat. No. 7524), 1% endothelial cell growth supplement (ScienCell Research Laboratories, Cat. No. 1052), 100 mg/mL streptomycin and 100 U/mL penicillin (ScienCell Research Laboratories, Cat. No. 0503). Cells between the 5th and 10th passage were used in this study and their viability was assessed through Trypan Blue Exclusion Test (ThermoFisher Scientific), as reported by Strober W [61], before performing any experiment.

### 4.2. GBM Biopsies

GBM tumor biopsies (*n* = 28) and unaffected brain parenchyma (*n* = 20) were obtained, classified and stored as previously described [18]. Written informed consent was supplied by all the patients enrolled in this study, before surgery. The study was conducted following the declaration of Helsinki’s guideline and was approved by the ethical committee of Azienda Ospedaliero-Universitaria “Policlinico-Vittorio Emanuele”, Catania, Italy (project identification code: 166/2015/PO, 17 December 2015). Clinical data from patients enrolled in the study are summarized in Table 3.

### 4.3. Cloning and Transfection

WT circSMARCA5 was cloned in a pcDNA3 vector as previously described [8]. Mutated circSMARCA5 sequences were generated through PCR-based replacement as reported in Table 1. Briefly, PCR products, each comprising mutated sequences of two predicted SRSF1 BSs at once, were generated starting from the cDNA of WT circSMARCA5 by using primers listed in Table S1. PCR products were then digested by using restriction enzymes (RE) listed in Table S1 and ligated into the WT circSMARCA5 pcDNA3 vector (previously digested with the same RE), replacing the corresponding WT sequences and generating the three constructs named Mut1, Mut2 and Mut3. pcDNA3 vector was purchased from Invitrogen. pcDNA3-FLAG and FLAG-tagged SRSF1 vectors were gifts from Dr Karoline Ebbesen. U87MG and CAS-1 were transfected using Lipofectamine 2000 (Thermo Fisher Scientific, Waltham, MA, USA), according to the supplier’s protocol.

### 4.4. RNA Extraction, Northern Analysis and qRT-PCR

RNA extraction from cells, northern analysis and qRT-PCR were performed as previously described [8,18]. Collection of cell culture supernatants and extracellular RNA isolation were performed as described by Bakr and colleagues [62]. Primer and probe sequences used in this study are listed in Table S2.

### 4.5. RIP Assay

RIP and qRT-PCR data analyses were performed as described in our previous paper [18], with some modifications. Briefly, cell lysates, obtained from U87MG co-transfected with FLAG-tagged SRSF1 and either WT or each of the mutated circSMARCA5, were immunoprecipitated (IPed) by using 2.5 μg of mouse monoclonal ANTI-FLAG^®^ M2 antibody (Sigma-Aldrich, Cat. n. F1804-5MG) or mouse isotype control IgG (negative control) (Santa Cruz Biotechnology, Inc., Heidelberg, Germany, Cat. n. sc-2025). RNA was then isolated from each input and IPed fraction, retrotranscribed and amplified, accordingly. Data were analyzed as described by Ratnadiwakara and colleagues [63]. RIP methodology and data analysis are fully described in Supplementary Methods.

### 4.6. Cell Migration Assay

Cell migration was assayed using Oris™ Cell Migration Assay (Platypus Technologies, Madison, WI, USA), as previously described [64]. Briefly, 3 × 10^5^ (CAS-1) or 4 × 10^5^ (U87MG) cells/well were seeded in a six-well plate, grown for 24 h and transfected for other 24 h. Cells were then trypsinized and 4 × 10^4^ (CAS-1) or 3.5 × 10^4^ (U87MG) cells/well were seeded in a 96-well migration plate with the stoppers placed. After 24 h of growth in a 5% FBS medium, the stoppers were removed, except for the pre-migration reference (T0) wells, where they remained in place until the time of assay readout. The plates were incubated at 37 °C in a humidified 95% air and 5% CO_2_ atmosphere for 24 h (U87MG) or 32 h (CAS-1) to allow cell migration. Stoppers were then removed from the T0 wells and images were captured using a Fluorvert inverted microscope (Leitz, Wetzlar, Germany) and then imported into Image J software v. 1.51 (National Institutes of Health, Bethesda, Maryland, USA) for data analysis.

### 4.7. Tube Formation Assay

The ability of IM-HBMEC to migrate and generate capillary-like structures was assessed by using the Matrigel assay (BD, Franklin Lakes, NJ, USA). Fifty microliters of growth factor reduced Matrigel matrix were added to a 96-well plate and allowed to solidify at 37 °C for 30 min. CMs harvested from CAS-1 and U87MG 48 h after transfection were centrifuged at 1000× *g* for 5 min, filtered with a 0.2 μm filter and stored at −80 °C until use. For all conditions, CMs were used at 1:1 dilution with serum and endothelial cell growth supplement-free HBMEC medium. IM-HBMEC (10^4^ cells/well) were treated with CMs or 25 ng/mL recombinant human VEGF-A (VEGF-A165 isoform) (Peprotech, Rocky Hill, NJ, USA) for 6 h (37 °C, 5% CO_2_) to allow the formation of tube-like structures. Photographs at 40× magnification were obtained using an inverted Leica DM IRB microscope equipped with a charge-coupled device (CCD) camera, as previously described [65]. Tube formation was assessed by measuring the total tube length and counting tubule branch points. The latter can be defined as cell junctions with at least three tubules (sections of the tube structure where three or more tubes converge). Angiogenesis Analyzer tool for ImageJ software (National Institutes of Health) was used for quantifications.

### 4.8. In Silico Analysis

Experimentally validated SRSF1-associated RNA motifs (*n* = 445) were downloaded from ATtRACT database [66] and used as a training set for the FIMO tool (within MEME Suite v. 5.3.0, National Institutes of Health), an algorithm that scans a set of sequences for occurrences of known motifs [67]. The FIMO tool was used to calculate the occurrences of validated SRSF1-associated RNA motifs within the predicted SRSF1 BSs lying in circSMARCA5 sequences, setting significant *p*-values < 10^−4^.

### 4.9. Statistical Analysis

Statistical significance was assessed as described within the text and figure legends, setting the *p*-value cut-off ≤ 0.05. Values are represented as the mean ± SD of at least three independent biological replicates (*n* = 3 or more), as specified in each figure legend. SPSS v. 23 software (SPSS, Chicago, IL, USA) was used to perform statistical analysis.

## 5. Conclusions

In conclusion, our data indicate GAUGAA as the RNA motif responsible for the interaction between circSMARCA5 and SRSF1 as well as for the circSMARCA5-mediated control of GBM cell migration and angiogenic potential.

## Figures and Tables

**Table 1:** Sequence, genomic localization and nucleotide (nt) position of mutated Serine and Arginine Rich Splicing Factor 1 (SRSF1) binding sites (BSs) within Mut1, Mut2 and Mut3 circSMARCA5. Prediction of SRSF1 BSs was performed by RBPMap (http://rbpmap.technion.ac.il/).

#	Mutated circSMARCA5 ID	Mutated Sequence of Predicted SRSF1 BS	Predicted SRSF1 BS Genomic Localization (GRCh38/hg38 Assembly)	Localization within circSMARCA5 (nt)
1	Mut 1	TCTACTT	chr4:143543546–143543552	38–44
2		ACTACTTCTA	chr4:143543615–143543624	107–116
3	Mut 2	CGATCCA	chr4:143543627–143543633	119–125
4		TCCACGT	chr4:143543645–143543651	137–143
5	Mut 3	TCTACTTACT	chr4:143543860–143543869	157–166
6		GCTTCCTCTTCTG	chr4:143543942–143543954	239–251

**Table 2:** Experimentally validated SRSF1 binding motifs significantly occurring within predicted SRSF1 BSs.

#	SRSF1 Binding Motif	*p*-Value	*q*-Value	Mutated circSMARCA5
1	UGAAGAU	6 × 10^−5^	0.0011	Mut1
2	UGAUGAA	6 × 10^−5^	0.0011	Mut1
3	GAAGGA	6 × 10^−5^	0.0011	Mut3

**Table 3:** Demographic data including sex and age of the study group.

Sample	*n*	Mean Age (Years ± StdDev)	Sex	
			Male	Female
Fresh frozen GBM biopsies	28	62.9 ± 11.1	14	14
Fresh frozen unaffected brain parenchyma	20	64 ± 10.3	8	12

## Data Availability

Not applicable.

## Supplementary Materials

The following are available online at https://www.mdpi.com/1422-0067/22/4/1678/s1.
