# Supplementary material for: The GAUGAA Motif Is Responsible for the Binding between circSMARCA5 and SRSF1 and Related Downstream Effects on Glioblastoma Multiforme Cell Migration and Angiogenic Potential
Source: Int J Mol Sci. 2021 Feb 7;22(4):1678. doi: 10.3390/ijms22041678 (PMC7915938; doi:10.3390/ijms22041678)
Supplement: Supplementary file 1 [file ijms-22-01678-s001.pdf]

Supplementary Tables

Table S1. Primer sequences (Fw = forward, RW = reverse) and restriction enzymes (RE) used to clone WT and mutated circSMARCA5.

Primer name	RE	Cloned circSMARCA5	Primer Sequence
circSMARCA5 FW (BamHI)	Bam H1	WT, Mut1	TCAGggatccTGTGTAAAGTTTGGCCTAGGTG
circSMARCA5 RW (ClaI)	ClaI	Mut1	atgaATCGATTAGAAGTAGTGTGATCTCACTTTCCTTTGAAGCAAACACATGTGTTGCTCCATGTCTAATCATTTGAAGCATAAGTAGATTCCCAATTTTGTTCAGATT
circSMARCA5 FW (ClaI)	ClaI	Mut2	atgaATCGATCCAATTTTGGAAAGTCCACGTAAGAAGgtgagatgtagat
circSMARCA5 FW (XbaI)	XbaI	Mut3	atgaTCTAGACTGCAGTCTACTTACTAAAGCTCTCCAAGATGGGCGAAAGTTCACTTAGAAACTTTACAATGGATACAGAGTCAAGTGTTTATAACTTGCTTCCTCTTCTGTATAGAGAA
circSMARCA5 RW (ApaI)	ApaI	WT, Mut2, Mut3	TCAGgggcccGGAACAACTGTTAAATGAGGGAGA

Table S2. Sequence, amplicon size, and annealing temperature of RT-PCR primers and probe used in this study.

Transcript name	Primer or probe sequence	Ta	Amp length (bp)
GAPDH	Fw: 5'-GTCAGCCGCATCTTCTTTTG-3'<br>Rev: 5'-GCGCCCAATACGACCAAATC-3'	60 °C	100
TBP	Fw: 5'-ACTTGACCTAAAGACCATTGCA-3'<br>Rev: 5'-GGCTCTCTTATCCTCATGATTACC-3'	60 °C	90
Total VEGFA	Fw: 5'-GCACCCATGGCAGAAGG-3'<br>Rev: 5'-CTCGATTGGATGGCAGTAGCT-3'	60 °C	90
VEGFA Iso8a	Fw: 5'-TTCCTGCAAAAACACAGACTCGC-3'<br>Rev: 5'-TCACCGCCTCGGCTTGTCACAT-3'	60 °C	88
VEGFA Iso8b	Fw: 5'-TTCCTGCAAAAACACAGACTCGC-3'<br>Rev: 5'-TCAGTCTTTCCTGGTGAGAGATCTGCA-3'	60 °C	88
circSMARCA5 (endogenous; WT; Mut1; Mut2; Mut3)	Fw: 5'-ACAATGGATACAGAGTCAAGTGTT-3'<br>Rev: 5'-CACATGTGTTGCTCCATGTCT-3'	60 °C	143
circSMARCA5 (probe)	GTGATCTCACTTTCCTTTGAAGCAAACACATGT GTTGCTCCATGTCTAATCATTTGAAGC		

Supplementary Figures

A

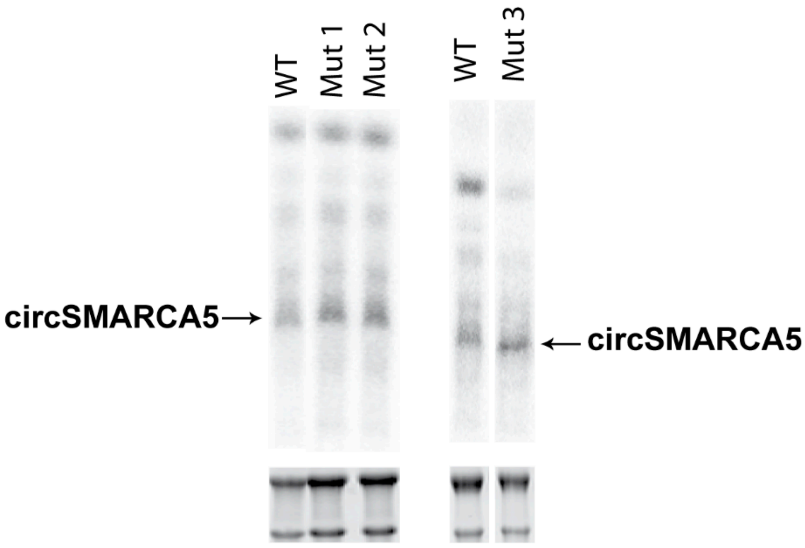

B

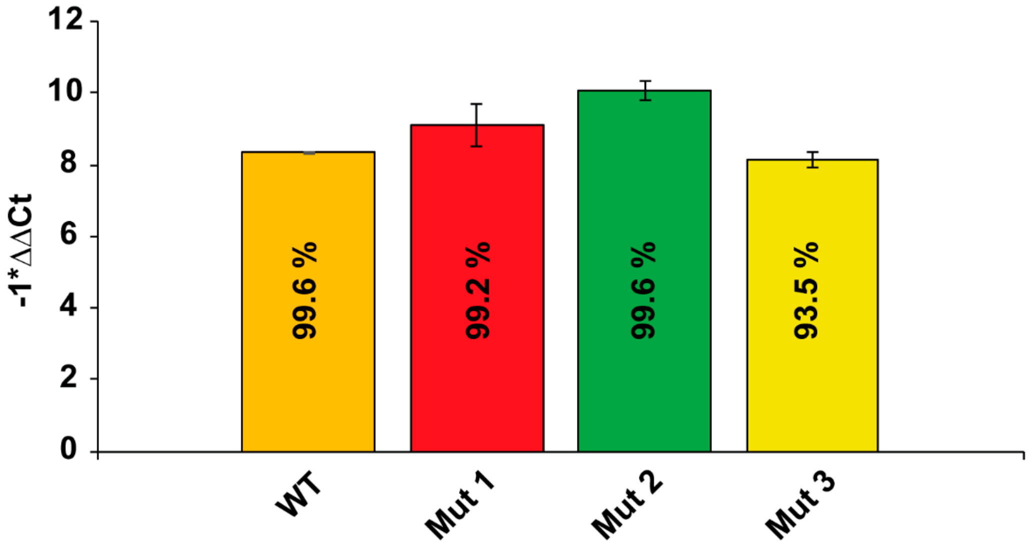

Figure S1. Northern blotting and qRT-PCR of WT and mutated circSMARCA5. (A) Northern blotting of WT and mutated circSMARCA5. A probe recognizing a region common to endogenous and transfected WT, Mut1, Mut2 and Mut3 circSMARCA5 has been used during hybridization. (B) qRT-PCR data of WT and mutated circSMARCA5. The bar graph represents the relative expression (reported as mean ± Standard Deviation of -1*ΔΔCt values) of circSMARCA5 in the different experimental conditions *vs* untransfected cells (endogenous circSMARCA5). TATA-box binding protein (TBP) mRNA was used as endogenous control transcript. Numbers shown within each bar are indicative of the percentage of transfection efficiency.

A

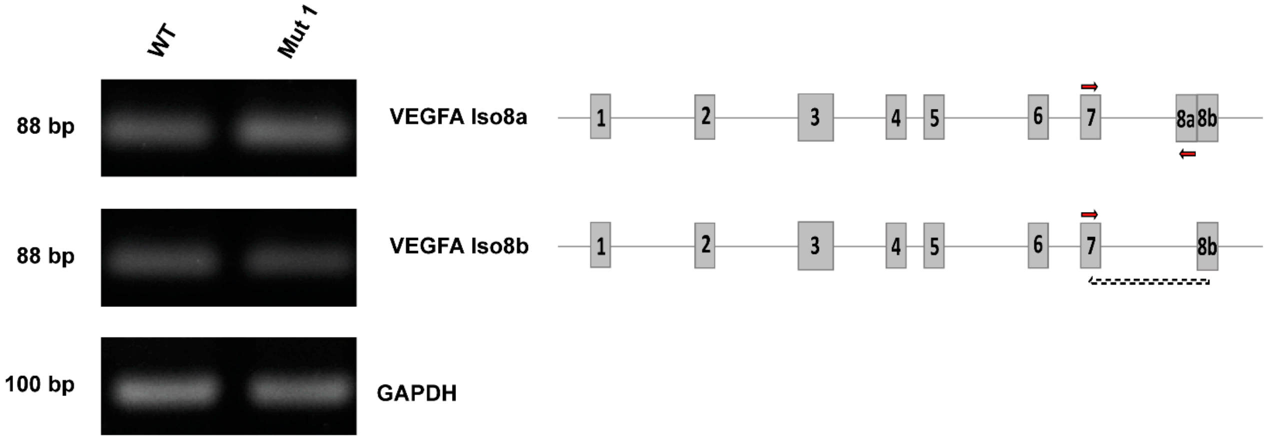

B

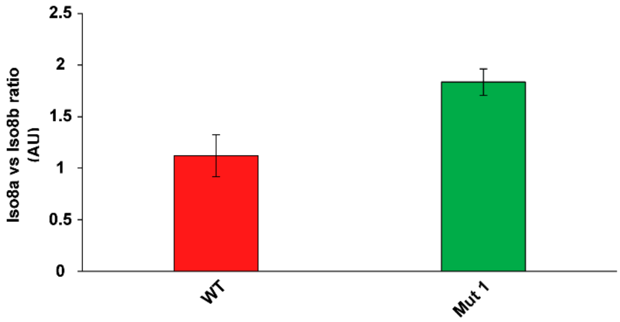

Figure S2 Qualitative PCR of Iso8a and Iso8b VEGFA mRNA isoforms in CAS-1 transfected with WT and Mut1 circSMARCA5. (A) Representative image of endpoint PCR products run over an agarose gel (1.8%). Beside gel electrophoresis bands the amplimer length (bp) (on the left) and the schematic diagrams of each amplified isoform (on the right) are reported. Introns and exons are represented as lines and boxes, respectively; the exon number is shown within each box. Forward and reverse primers are represented as rightwards and leftwards arrows, respectively. Primer whose sequence spans two different exons is represented as dashed arrow. (B) Bar graphs representing the ratio between Iso8a *vs* Iso8b VEGFA mRNA isoforms in CAS-1 transfected with WT and Mut1 circSMARCA5. Data are reported as mean ± standard deviation of three independent experiments. AU = arbitrary units (quantitative data estimated from densitometric analysis of the gel electrophoresis bands, performed through ImageJ). *GAPDH* was used as endogenous control. * p-value < 0.05, ** p-value < 0.01 (n = 3, Student's t-test vs WT).

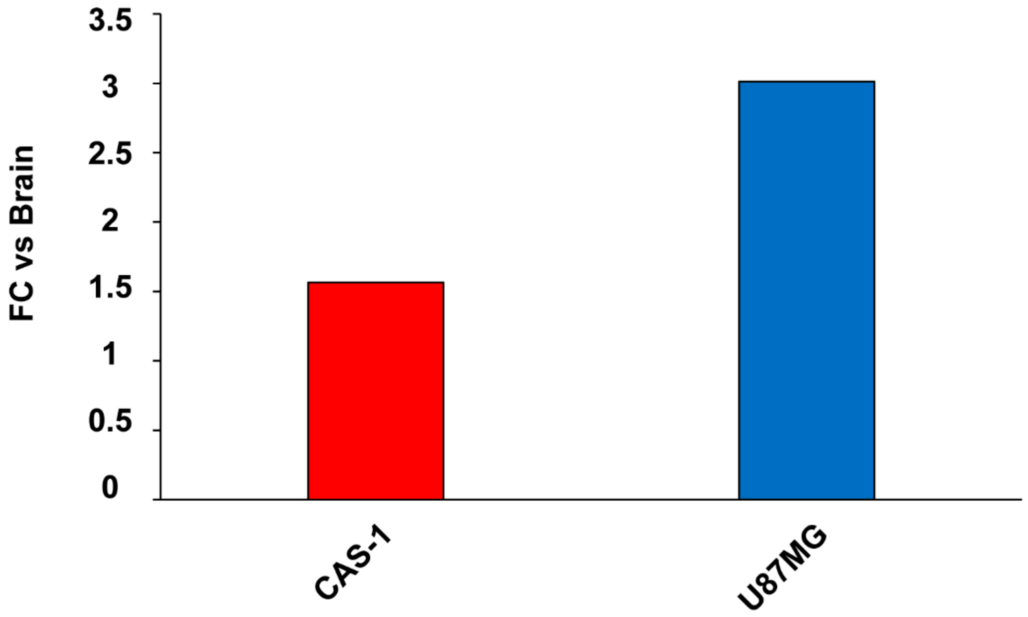

Figure S3. DHX9 mRNA expression in CAS-1 and U87MG. Expression of DHX9 mRNA evaluated by qRT-PCR in CAS-1 and U87MG. Data are expressed as fold-change (FC) *vs* unaffected brain (FirstChoice® Human Brain Reference RNA (Ambion, Austin, TX, USA). TATA-box binding protein (TBP) mRNA was used as endogenous control transcript.

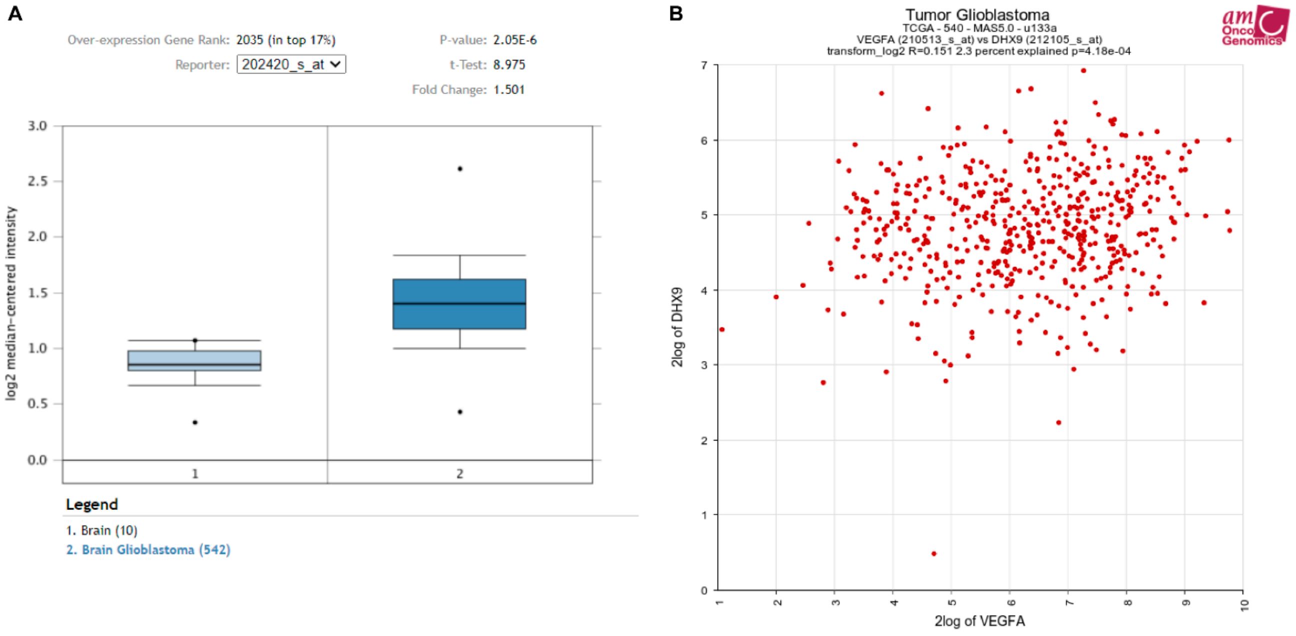

Figure S4. Gene expression analysis from TCGA data. (A) Expression of DHX9 mRNA in a cohort of 542 GBM and 10 normal brain (TCGA data, from Oncomine (https://www.oncomine.org/)). (B) Correlation between DHX9 and VEGFA expression in a cohort of 540 GBM (TCGA data, from R2Genomics (https://hgserver1.amc.nl/cgi-bin/r2/main.cgi)).

Supplementary Materials and Methods

RIP

For each experimental condition (FLAG-tagged SRSF1 + circSMARCA5 WT; FLAG-tagged SRSF1 + circSMARCA5 Mut 1, FLAG-tagged SRSF1 + circSMARCA5 Mut 2, FLAG-tagged SRSF1 + circSMARCA5 Mut 3) DCt_s were calculated as $Ct_{IPed\ (either\ FLAG\text{-}tagged\ SRSF1\text{-}IPed\ or\ IgG\text{-}IPed)} - Ct_{INPUT}$. Then DDCt was calculated as $DCt_{FLAG\text{-}tagged\ SRSF1} - DCt_{IgG\text{-}IPed}$. Fold change was then calculated as $2^{-\Delta\Delta Ct}$. FC of *GAPDH* mRNA was set to one and FC of circSMARCA5 was calculated accordingly. FC data were corrected both for the total amount of SRSF1 in INPUT and for the amount of IP_{ed} SRSF1 expression further to circSMARCA5 transfection efficiency (when co-transfected together with FLAG-tagged SRSF1), for each experimental condition.

Reverse transcription and endpoint PCR

Potential genomic DNA contamination was removed from the extracted RNA by DNase I Amplification Grade (ThermoFisher Scientific). 1 µg of DNase treated RNA was then reverse transcribed using SuperScript™ II Reverse Transcriptase (ThermoFisher Scientific) and random hexamer primers (Sigma-Aldrich), following manufacturer's instructions. cDNA was amplified using Platinum™ Taq DNA Polymerase (ThermoFisher Scientific) in a Biometra Personal Cycler thermocycler (Analytik Jena AG, Jena, Germany, EU). The linear range of amplification had been previously determined for each primer pair starting from serially diluted cDNA, amplified over different cycles of the PCR, so densitometric band analysis was performed during the exponential phase of the reaction, in non-saturated condition (data not shown). PCR products were resolved on a 1.8% agarose gel stained with SYBR™ Safe DNA Gel Stain (ThermoFisher Scientific). Agarose gel were then visualized through iBright FL1500 imager (ThermoFisher Scientific) and densitometric band analysis was performed through ImageJ software v. 1.51. Primers used to amplify GAPDH (reference gene) and specific pro-angiogenic (VEGFA Iso 8a) and anti-angiogenic (VEGFA Iso8b) isoforms were synthesized by Eurofins Genomics (Eurofins Genomics, Germany, EU): their sequences are listed in Table S2.
